# Pathophysiological Roles of Cytokine-Chemokine Immune Network

**DOI:** 10.1155/2014/615130

**Published:** 2014-09-11

**Authors:** Jong-Young Kwak, Mizuko Mamura, Jana Barlic-Dicen, Evelin Grage-Griebenow

**Affiliations:** ^1^Department of Biochemistry, School of Medicine and Immune-Network Pioneer Research Center, Dong-A University, 32 Daesin-gongwon-ro, Seo-gu, Busan 602-714, Republic of Korea; ^2^Department of Molecular Pathology, Tokyo Medical University, 6-1-1 Shinjuku, Shinjuku-ku, Tokyo 160-8402, Japan; ^3^Department of Internal Medicine, Kyungpook National University, 50 Samduk-2ga, Jun-gu, Daegu 700-721, Republic of Korea; ^4^Cardiovascular Biology, Oklahoma Medical Research Foundation and Department of Cell Biology, University of Oklahoma Health Sciences Center, 825 N.E. 13th Street, Oklahoma City, OK 73104, USA; ^5^Institute for Experimental Medicine, Inflammatory Carcinogenesis, UK S-H, Kiel Campus, Arnold-Heller-Straße 3, Building 17, 24105 Kiel, Germany

Cytokines are small proteins or glycoproteins, and chemokines form a group of smaller cytokines with chemotactic properties that are produced by a variety of cells. Cytokines and chemokines exert crucial roles in the development, homeostasis, activation, differentiation, regulation, and functions of innate and adaptive immunity. Excessive and/or inappropriate production and actions of cytokines and chemokines are involved in the pathogenesis of infection, inflammation, allergy, autoimmune diseases, and immune-related diseases, such as diabetes, atherosclerosis, rheumatic arthritis, and cancer. Understanding the underlying mechanisms by which cytokines and chemokines exert their functions will lead to development of therapeutics for immune-related diseases. In this special issue, the articles and reviews discuss recent findings that highlight the pathophysiological role of cytokine and chemokines.

Effector and memory T cells are phenotypically and functionally heterogeneous. Upon antigenic stimulation, naïve CD4^+^ T cells become effector Th1, Th2, or Th17 cells or regulatory T cells (Tregs). Effector and regulatory T cell subsets are generated in the presence of specific cytokines such as IL-12 and TGF-*β*, respectively. Transcription factors initiate and stabilize commitment toward the Th1 or Th2 lineage. T-bet (the T-box protein expressed in T cells) is the master regulator of Th1 differentiation [1]. A review article by S. Oh and E. S. Hwang summarizes the current state of knowledge regarding the molecular mechanisms that underlie the multiple roles played by T-bet in T helper cell development and fine-modulation of IL-2 production in Th1 cells. Interestingly, IFN-*γ* stimulation of dendritic cells (DCs) might have an equally important role in generating different effector T cells [2]. In this special issue, A. Visperas et al. describe several cytokines that are able to influence the generation of effector T cells by directly affecting T cells as well as targeting non-T cells. Inflammatory cytokine signaling also plays an important role in the pathogenic conversion of natural Tregs. The article by R. Takahashi and A. Yoshimura is a review in which the possibility is proposed that suppressor of cytokine signaling 1 (SOCS1) may protect Tregs from harmful effects of inflammatory cytokines, and SOCS1 upregulation maintains Treg functions. SOCS1 may be important in the pathogenesis of systemic lupus erythematosus through Treg plasticity, because SOCS1-deficient T cells induce lupus-like autoimmunity [3].

Both tumor-antagonizing and tumor-promoting inflammatory cells can be found in most neoplastic lesions. Inflammation can increase the risk of cancer by providing bioactive molecules from cells that infiltrate the tumor microenvironment, including cytokines and chemokines [4]. G. Landskron et al. review the roles of several inflammatory mediators, including TNF-α, IL-6, TGF-*β*, and IL-10, in events of carcinogenesis, such as their capacity to generate reactive oxygen and nitrogen species; their potential mutagenic effect; and involvement in mechanisms for epithelial mesenchymal transition, angiogenesis, and metastasis. They also provide an in-depth analysis of the participation of cytokines in cancer that is attributable to chronic inflammatory diseases, such as colitis-associated colorectal cancer and cholangiocarcinoma.

Recent studies have shown that the circadian clock is responsible for the temporal dynamics of the immune system [5]. For example, joint stiffness and secretion of inflammatory cytokines in rheumatoid arthritis (RA) patients are influenced by the diurnal rhythm and peaks in the morning. RA is a devastating autoimmune disease that is characterized by progressive bone destruction and it was found that the circadian clock not only impacts arthritic symptoms but is also involved in the pathogenesis of RA [6]. Proinflammatory cytokines, such as IL-1, IL-6, IL-8, IL-11, IL-17, and TNF-α, are known to be osteoclastogenic [7]. In this special issue, S. M. Jung et al. discuss the osteoclastogenic role of the proinflammatory cytokines and immune cells in the pathophysiology of RA. Moreover, a review by A. Nakao summarizes recent advances regarding the emerging role of the circadian clock as a novel regulator of cytokines. K. Yoshida et al. describe the link between the circadian clock and inflammation, focusing on the interactions of various clock genes with the immune-pathways underlying the pathology of RA. In another article, Y. Nakamura et al. demonstrate that mechanical disruption of the suprachiasmatic nucleus (SCN) of the hypothalamus resulted in the absence of time of day-dependent variation in anaphylactic reaction in mice. These articles provide evidences that daily variations in cytokine levels are related to the pathophysiology of immune diseases.

Obesity, insulin resistance, and atherosclerosis are chronic inflammatory processes that are affected by the activation of innate and adaptive immunity [8, 9]. With regard to the role of inflammation of adipose tissue in obesity and obesity-related diseases, aberrant production of adipokines, cytokines, and chemokines in adipose tissue leads to inflammation in the tissue. A review article on this topic by L. Yao et al. covers the chemokine system and signaling in the development of obesity, insulin resistance, and plaque formation.

A clinical study by K. Theodoraki et al. demonstrates that IL-10 levels were elevated in patients that were subjected to major abdominal surgery procedures with a more liberal red blood cell transfusion strategy. Their results indicate that IL-10 may be an important factor in transfusion-associated immunomodulation. Expression of proinflammatory cytokines and chemokines has also been reported in patients with chronic venous insufficiency, such as varicose veins [10]. V. Tisato et al. demonstrate that EGF, PDGF, and RANTES were increased in varicose veins compared with general circulation, and a patient who exhibited recurrence of the disease 6 months after surgery showed higher levels of these factors compared with nonrecurrent patients. Therefore, V. Tisato et al. suggest that EGF, PDGF, and RANTES can be used as sensitive biomarkers of chronic venous insufficiency. Finally, J. Sturgill et al. examine the correlation of cytokine production in plasma of patients with fibromyalgia and they suggest that suppression of Th2 cytokines is related to immune dysregulation in patients with fibromyalgia.
